# Abducens nerve palsy as a postoperative complication of minimally invasive thoracic spine surgery: a case report

**DOI:** 10.1186/s12893-016-0162-1

**Published:** 2016-07-13

**Authors:** Luiz Henrique Dias Sandon, Gun Choi, EunSoo Park, Hyung-Chang Lee

**Affiliations:** Neurosurgery Resident at Hospital das Clinicas de São Paulo, São Paulo, Brazil; International Spine Surgery Fellow, Pohang Wooridul Hospital, Pohang, South Korea; Neurosurgeon/Spine Surgeon and Medical Director, Pohang Wooridul Hospital, Pohang, South Korea; Neurosurgeon/Spine Surgeon and Consultant, Pohang Wooridul Hospital, Pohang, South Korea; Thoracic Surgeon/Consultant at Busan Wooridul Hospital, Busan, South Korea; Department of Neurosurgery, Hospital da Clinicas FMUSP, Rua Oscar Freire, 1811, ap 113 Cerqueira Cesar, Sao Paulo Brazil

**Keywords:** Cranial nerve palsy, Cerebrospinal fluid leakage, Intracranial hypotension, Thoracic disc herniation, Minimally invasive

## Abstract

**Background:**

Thoracic disc surgeries make up only a small number of all spine surgeries performed, but they can have a considerable number of postoperative complications. Numerous approaches have been developed and studied in an attempt to reduce the morbidity associated with the procedure; however, we still encounter cases that develop serious and unexpected outcomes.

**Case Presentation:**

This case report presents a patient with abducens nerve palsy after minimally invasive surgery for thoracic disc herniation with an intraoperative spinal fluid fistula. A literature review of all cases related to this complication after spine surgery is included.

Despite the uncommon nature of this type of complication, understanding the procedure itself, the principle occurrences and outcomes following the procedure, the physiopathogical features of abducens nerve palsy, and the possible adverse effects of spinal surgery, including minimally invasive procedures, can enable an early diagnosis of complications and facilitate the procedure.

**Conclusions:**

In spite of being very rare and multifactorial, uni- or bilateral abducens nerve paralysis carries significant morbidity and can occur as a postoperative complication after conventional or minimally invasive spine surgery. This condition requires an accurate diagnosis and adequate multidisciplinary follow up.

## Background

Medullary lesions are less likely to occur in the region along the anatomical structure of the thorax. In this area, the spine is protected by the large muscles and rib cage, and it has reduced mobility compared to the cervical or lumbar portions. However, due to the reduced diameter of the spinal canal, small osseous deformities or disc protrusion can result in significant clinical symptoms [1].

The incidence of symptomatic disc herniation in the thoracic spine is estimated to be 1 in 1,000,000 in the general population [2], and its etiology has not been well established. The symptoms can vary from mild back pain to moderate neurological deficits (sensory or motor) to severe paraparesis with or without sphincteral alterations. Surgery is the treatment of choice for more severe cases that do not respond to clinical treatment or are accompanied by progressive neurological deficits [2, 3].

Surgical procedures for discectomy in the thoracic region constitute only 0.15 to 4 % of all surgical procedures for discal hernia, and the index of reported complications varies from 11 to 21 % [2].

Different surgical approaches have been proposed for this region. Posterior approaches, such as laminectomy with or without transversectomy, have been found to be minimally effective, with insufficient decompression and a high risk of complications [4]. Anterior approaches seem to improve the symptomology, but have a high rate of postoperative complications [5]. In recent years, the use of minimally invasive techniques has been proposed, including videothoracoscopy; however, the results of videothoracoscopy are not well established, due to a lack of cases and the surgeons’ learning curve for the endoscopic technique, which is further hampered by the small number of total cases [3]. Alternative minimally invasive techniques then arose, such as lateral retropleural transthoracic minimally invasive access, described by Berjano et al. for discectomy and decompression of the thoracic spine, which involves less invasive access and does not require collapsing the lungs or microsurgical decompression techniques. This technique produced good results in the short-term, but well-established studies of the long-term outcome are lacking [3, 6–8].

We describe a case of spinal cord compression with disc herniation that was treated with a minimally invasive surgical procedure at the Spine Surgery Service of the Pohang Wooridul Hospital and had an unusual progression.

## Case Presentation

The patient is a 47-year-old woman with a history of Type II diabetes mellitus, diagnosed 1 year prior. At the initial assessment, she was using oral hypoglycemic medication and achieving good control of her blood glucose level. Her condition began with back pain radiating to her lower limbs, more severely on the right side, and difficulty walking.

After 2 months, her condition worsened progressively and did not respond to conservative treatment and symptomatic medicine. She came to our clinic with paraparesis of the lower limbs (strength grade II) that was worse in the proximal left leg and associated with urine retention.

**Fig. 1:**
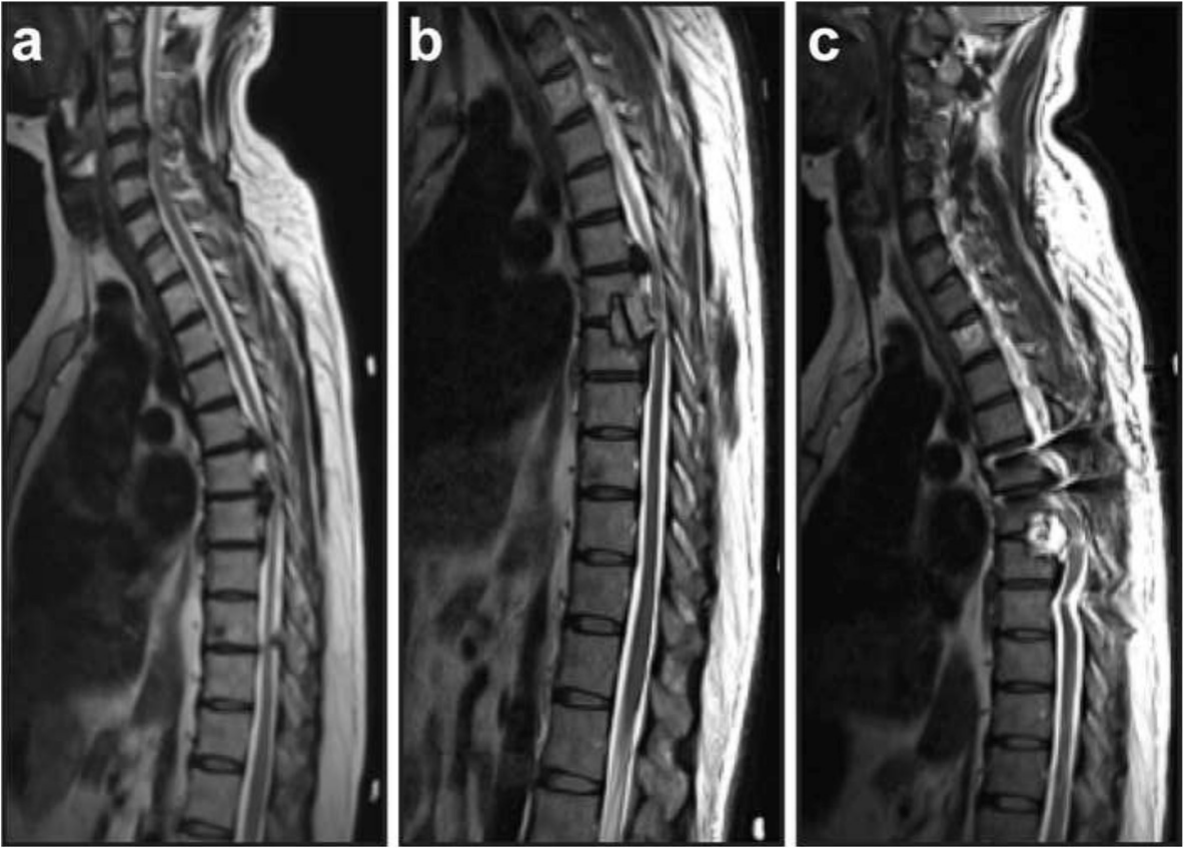
Magnetic resonance imaging (MRI) of the thoracic spine (T2). **a** The preoperative MRI (04/09/2015) **b** 1st MRI scan post-surgery (first surgery - 14/05/2015), **c** Post- operative MRI after the second surgery (second surgery - 20/04/2015)

The patient underwent magnetic resonance imaging (MRI) of the spine, which showed stenosis at the thoracic level with osteophytes in 3 thoracic vertebrae (T5/T6/T7), disc herniation at T5/6 – T6/7, and regional ligament thickening (ossification of the posterior longitudinal ligament; OPLL) affecting the spinal canal, with signs of myelopathy (Fig. 1a).

We opted to perform surgery, and we performed a microsurgical discectomy with minimally invasive left transthoracic access, with a partial posterior corpectomy of T6/7 and placement of an autologous bone graft. During the operation, we had difficulty removing the disc fragment due to its adherence in the posterior portion of the spinal cord and calcification of the local structures (vertebral disc and posterior longitudinal ligament). This caused a dural tear with visible spinal fluid leakage. Primary suturing of the dura mater was not possible; however, the fistula was promptly corrected with a synthetic patch of collagen and fibrinogen (Tachocomb®), without any visible residual fluid leakage during the surgery. A chest tube connected to a waterseal and an external lumbar drain were placed. It is important to emphasize that in cases of CSF leakage, the chest tube should only be placed to waterseal and no suction or negative pressure should be used, as was the case in this report

In the immediate postoperative period, the patient reported little improvement from her initial condition, with continued back pain and weakness in the lower limbs. However, the postoperative wound appeared to be healing well and the thoracic and lumbar drainage was adequate (slightly more than 150 ml/day and approximately 300 ml/day respectively). The first postoperative MRI scan showed that a residual bone fragment was in contact with the anterior portion of the spinal cord, and signs of edema and myelopathy were apparent as well. We did not observe any epidural, subcutaneous, or intracavity collections in the thorax (Fig. 1b).

Considering the lack of clinical improvement and the postoperative image showing unsatisfactory decompression of the spinal canal, on the 4th postoperative day another approach to the thoracic spine was deemed necessary. A revision of the previous discectomy was then performed with additional removal of the posterior bony portions of the vertebral bodies of T6 and T7 and repositioning of the previous bone graft, complemented by a laminectomy of T6/T7/T8 and arthrodesis with posterior fixation (using rod and pedicle screws) from T5 to T8. During this second surgery, neither dural lesions nor fluid leakage were observed.

In the immediate postoperative period after the second surgery, the patient reported an improvement in motor function and significant pain relief. The control MRI three days after the revision surgery showed satisfactory decompression of the thoracic spine and good positioning of the instrumentation devices. A small epidural hyperintense collection of fluid was noted in the anterior portion of the spine at T6-T7 (Fig. 1c).

The patient remained on bed rest as advised by the medical team, due to the treatment of the CSF leakage during the first surgical procedure (lumbar drain) and experienced progressive improvement in her pain level and neurological deficits. The chest tube was removed on the 9th postoperative day, following a consultation with the thoracic surgeon. Removal was based on criteria established in the literature for chest tube removal: (1) liquid drainage flow less than 150 ml/day; (2) 12 to 24 h after the air leak ceased; (3) resolution of pleural disease; (4) placement no longer than 10 days, even when drainage from the pleural disease is unresolved; and (5) fully expanded lungs. After 12 days, the external lumbar drain was removed. The patient was encouraged to ambulate on the 14th day after the first surgery (10th day after the second surgery), at which time she reported sudden diplopia and complained of a moderate headache, without blurred vision.

**Fig. 2:**
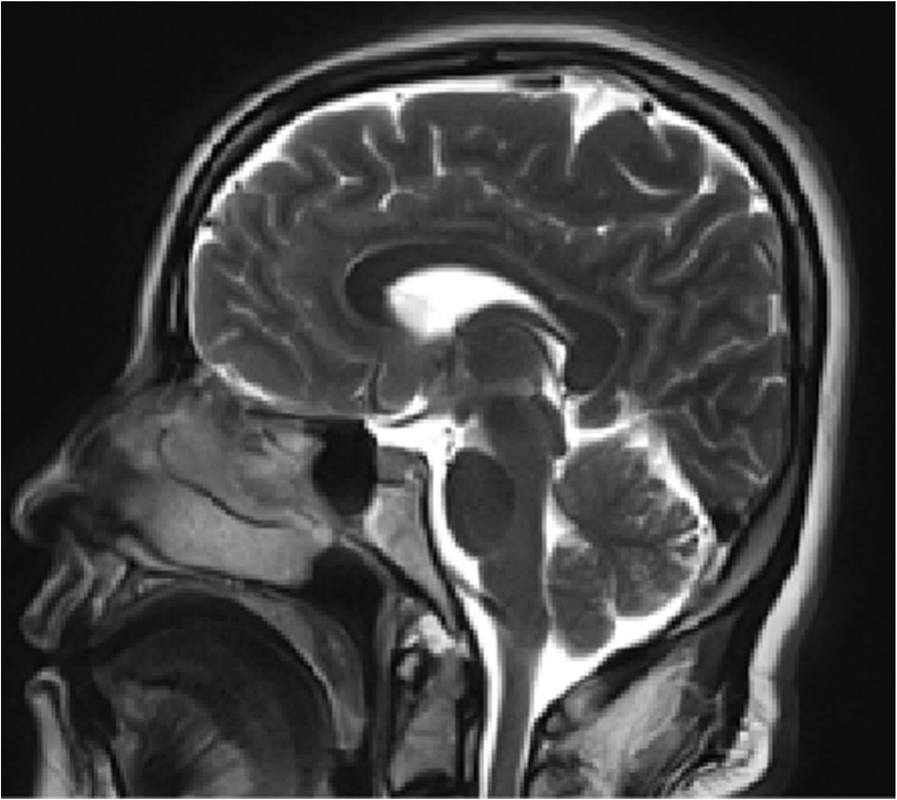
Sagittal T2 magnetic resonance imaging (MRI) of the brain (05/01/2015)

During the neurological examination, the patient was conscious and oriented but displayed a conjugate gaze to the left due to ophthalmoplegia of the lateral rectus muscle of the left eye (abducens nerve palsy), though the remainder of the exam was consistent with the patient’s baseline condition. An MRI of the brain did not show any signs of herniation or mass effects; however, we did observe a discrete diffuse ventricular reduction and an increased bilateral parietal subdural space, without bleeding or pneumoencephalus (Fig. 2).

We opted for clinical observation and local occlusion of the left eye for the patient’s comfort and radiological control of the case.

**Fig. 3:**
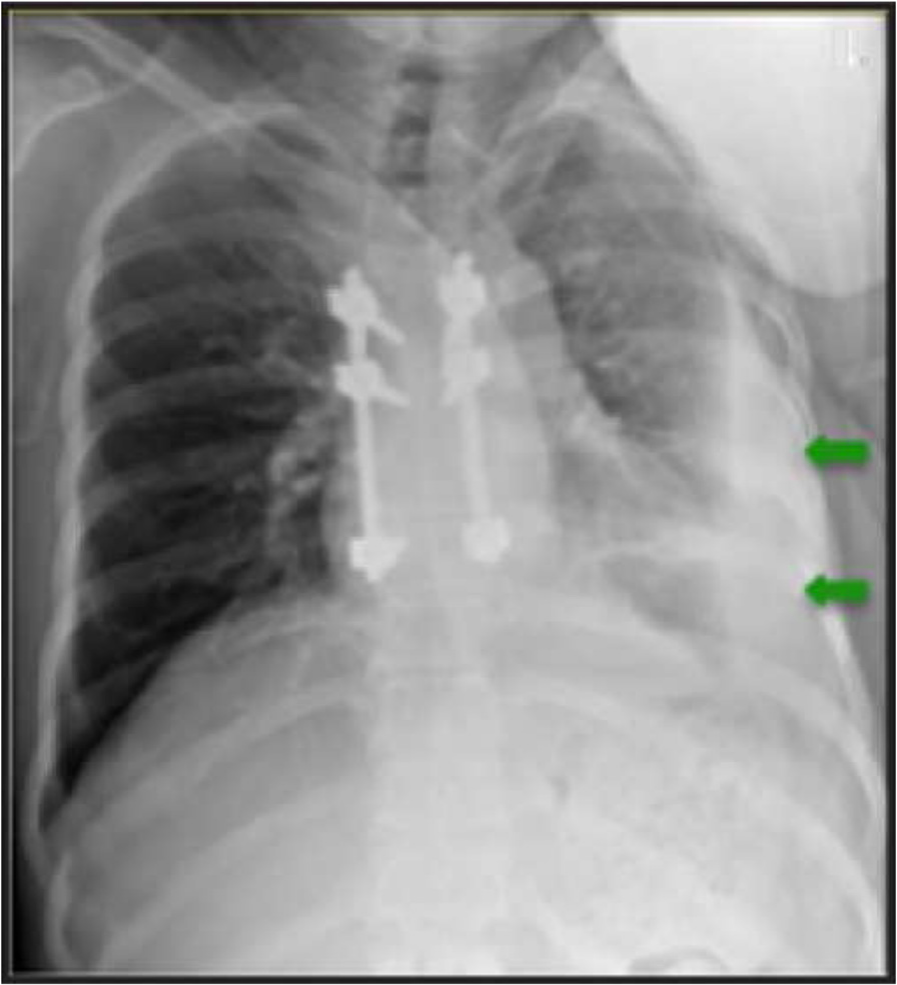
Chest radiograph in a lateral position, with pleural effusion in the left hemithorax (*arrows*)

On the 22nd postoperative day after the first surgery (18th day after the second), a pleural effusion was observed in the left hemithorax upon routine radiography, though the patient remained stable without any associated respiratory complaints (Fig. 3).

The initial planned treatment was unchanged. The patient was recommended for follow up including respiratory and motor physiotherapy.

**Fig. 4:**
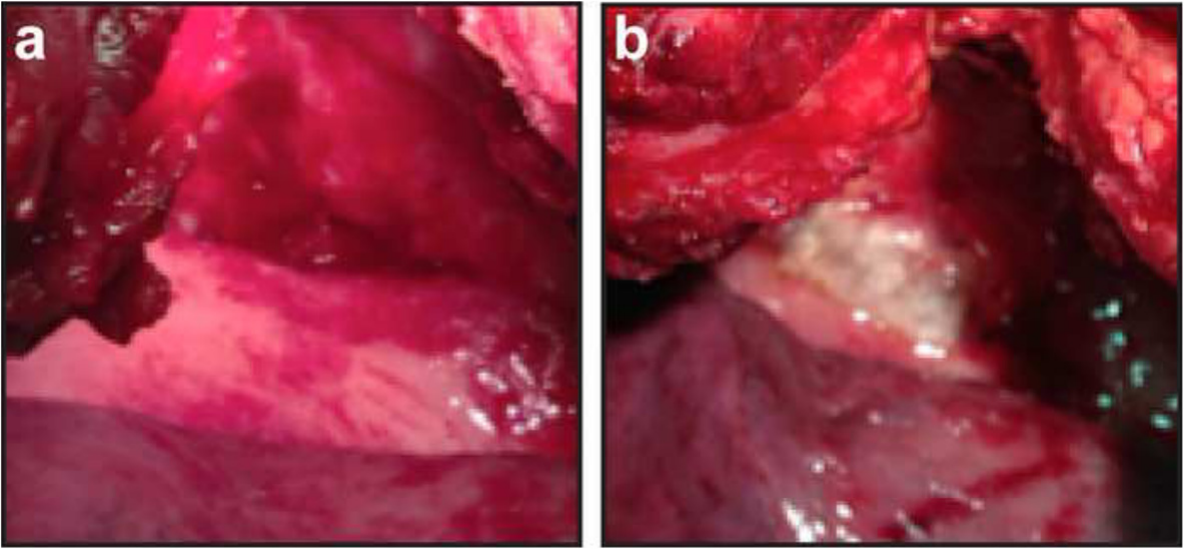
**a** Intraoperative image of the failure of the parietal pleura. In the lower part of panel **a**, the lung shifted to a previous position and then adhered to the aorta with fibrosis. The parietal pleura, anterior part of the spine, and the hole that connected the anterior portion of the spinal cord with the intrathoracic space can be seen in panel **a**, **b** Final appearance, including the patch and sealant

The patient’s headache progressively worsened and the abducens nerve palsy continued; radiographs of the pleural effusion in the left hemithorax and the MRI of the skull were also unchanged. On the 27th postoperative day after the first surgery (23rd postoperative day after the second) we decided to again attempt to correct the spinal fluid leak. During the procedure, which involved collaboration with the thoracic surgeon, it was not possible to mobilize the aorta in a way that would enable visualization of the anterior portion of the spinal cord and its dura mater, due to fibrosis and adherence from the previous surgeries; thus, we could not locate the dural defect itself. Nevertheless, we could clearly see a parietal pleura defect that resulted in a direct connection between the intrathoracic space and the spinal cord (Fig. 4a). We decided to seal the thoracic cavity to decrease the negative pressure over the point of the fistulization and to place an external lumbar drain to make it possible to close the dura mater as a second objective. Sealing the parietal pleura was accomplished with 3 layers of fibrin sealant (Tisseeal ®), a fibrin patch, and fibrinogen (Tachocomb®) (Fig. 4b); a primary suture was not possible.

The chest drain remained connected to the waterseal, and external lumbar drainage was maintained with a controlled flow of 100 ml every 8 h.

One week after the final correction of the fistula, the chest tube was removed under the direction of the thoracic surgeon. The patient began to show improvement overall and recovery of left eye mobility.

## Discussion

### CSF leakage and abducens nerve palsy

Many postoperative complications have been associated with spine surgery, with the most feared being dural fistulas.

The risk of a dural lesion after thoracic spine access is well known and is often inevitable when there is disc herniation or posterior longitudinal ligament ossification and adherence to the dura mater [5].

Treatment of a thoracic spine dural tear is challenging, especially when considering the inherent negative pressure in the chest cavity, a factor that results in continuous drainage of the cerebrospinal fluid (CSF), making it difficult to close up the fistula. Thus, until there is a definite correction of the spinal fluid fistula, a significant and progressive loss of spinal fluid can occur. Ultimately, abundant CSF leakage can cause intracranial hypotension and lead to serious consequences for the patient [9].

Paralysis of the cranial nerves can be a subsequent complication of intracranial hypotension [10]. It should be noted, however, that it is very rare to see paralysis of the sixth cranial nerve as a complication of spine surgery [11]. Barsoum et al. described some cases of cranial nerve VI paralysis related to traction during the procedure [12], and Abd-Elsayed et al. [13] reported paralysis related to facial swelling caused by the intra-operative positioning of the patient. However, only 6 cases are described in the literature that directly relate abducens nerve palsy to postoperative spinal fluid leakage (Table 1). Of these cases, only 2 were subsequent to surgeries of the thoracic region, and our case is the first to report this complication after the use of a minimally invasive technique. In 3 of these previously reported cases, the spinal fluid fistula was corrected surgically; in 3 others, a conservative approach involving clinical treatment was chosen. All cases described had complete resolution of the cranial nerve paralysis after a time period varying from weeks to months after the surgery (minimum: 1 week, maximum: 12 months), with a shorter time related to the early surgical correction of the spinal fluid fistula.

**Table 1:** Reported cases of paralysis of the abducent nerve after intraoperative dural fistula from spinal surgery

Year	Journal	Author	Surgery	Patient gender	Age	Spine level	Diabetes	CSF Leakage	IV Palsy detection	Treatment	IV Palsy Remission
2003	J. Orthopaedic science	Nakagawa et al. [10]	Spinal tumor Resection	Female	22	Cervical	No	Not identified	3 days Postop	Conservative	1 year
2009	J. Korean Neur Society	Cho et al. [14]	Posterior Fusion	Male	61	Lumbo-Sacral	No	Not identified	2 days Postop	Conservative	5 weeks
2012	Spine	Thomas et al. [11]	Discectomy	Male	53	Lumbar	Yes	Identified	7 days Postop	Dural Repair	2 weeks
2012	Eur Spine Journal	Joo et al. [15]	Discectomy	Male	48	Lumbo-Sacral	No	Identified	3 days Postop	Dural Repair	1 week
2013	Eur Spine Journal	Khurana et al. [9]	Discectomy	Male	48	Thoracic	No	Identified	>3 weeks Postop	Dural Repair + Chest drain	3 months
			Discectomy	Male	46	Thoracic	No	Identified	"Few days" Postop	Conservative	5 months

### Physiopathology

A 2007 literature review by Zada et al. [14] found 29 studies comprising 42 patients with intracranial hypotension and involvement of the cranial nerve that resulted in ocular deficiencies. Of these, 83 % of patients had ophthalmoplegia associated with paralysis of the abducens nerve; 60 % had unilateral ophthalmoplegia and 24 % bilateral [14].

Although abducens nerve paralysis can be secondary to systemic diseases such as diabetes or primary pontine lesions (tumors and/or ischemia), it is generally associated with compression or traction effects directly on the nerve [15].

The sixth nerve innervates the ipsilateral lateral rectus, which abducts the eye, and has the longest subarachnoid course of all of the cranial nerves [16]. Axons of the abducens nerve emerge from the ventral aspect of the brain stem at the pontomedullary junction. The nerve runs rostrally and slightly laterally in the subarachnoid space of the posterior cranial fossa to pierce the dura at a point lateral to the dorsum sellae of the sphenoid bone. It continues forward between the dura and the apex of the petrous temporal bone, where it takes a sharp right-angled bend over the apex and enters the cavernous sinus. Within the cavernous sinus, the sixth nerve is situated lateral to the internal carotid artery and medial to cranial nerves III, IV, VIand V2. Continuing forward, the abducens nerve leaves the cavernous sinus and enters the orbit at the medial end of the superior orbital fissure. It is then encircled by the tendinous ring, which provides a point of origin for the four recti muscles of the eye. The nerve enters the deep surface of the lateral rectus muscle, which it innervates [17].

With this long trajectory apposed to the skull base, the nerve is subject to the mechanical forces of traction and compression. With the outflow of fluid from the intracranial compartment, there is a reduction in the support strength of the brain and the brain stem structures, resulting in the relative decay of these structures. Thus, even without signs of herniation, the nerve can be directly stretched at its origin or pressed against bony structures surrounding its trajectory. This mechanical alteration affects the conduction of nerve impulses, which consequently paralyzes the muscle that it innervates [15, 18, 19].

### Outcome

Because this trauma is essentially a mechanical alteration that does not affect the inner structure of the nerve, a favorable outcome is expected for this patient.

According to the literature, 100 % of the cases reporting alterations of the sixth cranial nerve in the postoperative period after spine surgeries experienced complete remission from paralysis and the return of normal movement of the eye, within variable lengths of time.

As previously described, early surgical treatment of spinal fluid fistulae seems to reduce the recovery time and improve the prognosis [15].

Ocular occlusion is the first choice for the treatment of acute diplopia and its associated discomfort. Other options include the injection of botulinum toxin into the lateral rectus muscle or corrective eye surgery. Although we do not know the time limit for undertaking remedial measures successfully, invasive procedures should be considered only after months without improvement of the patient’s condition [17].

## Conclusion

We have described a postoperative complication of spine surgery that, despite being very rare and seemingly benign, had evident symptoms that could result in significant morbidity for the patient. This is the first case to report this complication (abducens nerve paralysis) after a minimally invasive thoracic spine surgery. We wish to emphasize the importance of the diagnosis, physiopathological understanding, and early treatment of such complications. Even in this case, it is possible that the earliest invasive measures could have avoided these complications; however, the exact judgment of the best time to perform invasive techniques is a great challenge. Therapeutic procedures should be performed as soon as possible, even if the choice is the most invasive technique.. However, often both risks and benefits are apparent, and a dilema arises; for example, while the chest tube was useful for the diagnosis and treatment of early complications, it also can be an aggravating factor. The literature regarding this issue is still insufficient, requiring further studies to explore these alternatives.

We also stress the need for clinical follow up involving a multidisciplinary team for the evaluation, rehabilitation, and future treatment of the patient.

## Abbreviations

CSF, cerebrospinal fluid; MRI, magnetic resonance imaging
